# Binder-Free Anodes for Potassium-ion Batteries Comprising Antimony Nanoparticles on Carbon Nanotubes Obtained Using Electrophoretic Deposition

**DOI:** 10.1021/acsami.4c02318

**Published:** 2024-07-01

**Authors:** Xuan-Manh Pham, Syed Abdul Ahad, Niraj Nitish Patil, Maria Zubair, Misbah Mushtaq, Hui Gao, Kwadwo Asare Owusu, Tadhg Kennedy, Hugh Geaney, Shalini Singh, Kevin M. Ryan

**Affiliations:** Department of Chemical Sciences and Bernal Institute, University of Limerick, Limerick V94 T9PX, Ireland

**Keywords:** anode, potassium-ion batteries, nanocomposites, carbon nanotube, electrophoretic deposition

## Abstract

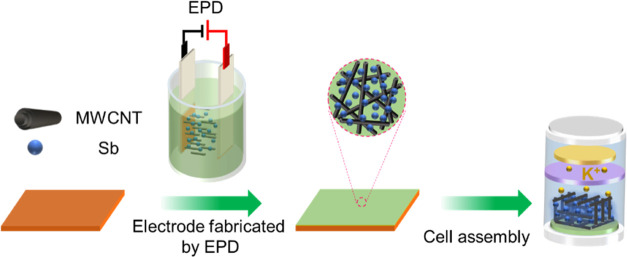

Antimony has a high theoretical capacity and suitable alloying/dealloying potentials to make it a future anode for potassium-ion batteries (PIBs); however, substantial volumetric changes, severe pulverization, and active mass delamination from the Cu foil during potassiation/depotassiation need to be overcome. Herein, we present the use of electrophoretic deposition (EPD) to fabricate binder-free electrodes consisting of Sb nanoparticles (NPs) embedded in interconnected multiwalled carbon nanotubes (MWCNTs). The anode architecture allows volume changes to be accommodated and prevents Sb delamination within the binder-free electrodes. The Sb mass ratio of the Sb/CNT nanocomposites was varied, with the optimized Sb/CNT nanocomposite delivering a high reversible capacity of 341.30 mA h g^–1^ (∼90% of the initial charge capacity) after 300 cycles at C/5 and 185.69 mA h g^–1^ after 300 cycles at 1C. Postcycling investigations reveal that the stable performance is due to the unique Sb/CNT nanocomposite structure, which can be retained over extended cycling, protecting Sb NPs from volume changes and retaining the integrity of the electrode. Our findings not only suggest a facile fabrication method for high-performance alloy-based anodes in PIBs but also encourage the development of alloying-based anodes for next-generation PIBs.

## 1 Introduction

Potassium-ion batteries (PIBs) have recently received revived interest as lower-cost alternative lithium-ion batteries (LIBs) for stationary storage in the near term and possible alternate to Li-ion in the long term for some transport applications in the event of scarce Li resources. This relates to the abundant reserves of potassium (K) (1.5 wt %) in the earth’s crust, the inexpensive cost of K compounds, and a comparable standard reduction potential of K^+^/K to Li^+^/Li.^1−5^ However, the large ionic radius of K^+^ (0.138 nm) as compared to Li leads to sluggish ionic transport during potassiation/depotassiation, resulting in poor material activation with fast capacity degradation.^1,2,6,7^ Therefore, seeking suitable anode materials and architectures capable of accommodating significant volume changes during K cycling with a high and stable capacity presents a huge challenge. Materials with alloying-type chemistry (e.g., Sb, Bi, Sn, etc.) have recently been viewed as one of the most promising anode material classes, primarily because of the low operating voltage, cost-efficiency, and high theoretical gravimetric capacities.^8−11^ Among them, Sb stands out as an especially attractive candidate for PIBs due to its low reaction potential, high electrical conductivity (2.56 × 10^6^ S·m^–1^), and high theoretical capacity (660 mA h g^–1^).^1,2,12−15^ Nevertheless, the huge volume change (∼407%) during the K-alloying reaction may lead to instability in the structure, which restricts the use of Sb-based material as an anode in PIBs.

To overcome these issues, a number of approaches have been proposed, involving advanced structural designs of Sb-based anodes, providing enough space for volume expansion and incorporating Sb with conductive carbonaceous materials (such as graphene, carbon nanotubes (CNT), etc.), which can relieve the stress induced by volume changes.^2,16−18^ Wang et al.^19^ reported an encapsulated Sb@carbon sphere network (Sb@CSN) electrode, maintaining a specific capacity of 504 mA h g^–1^ after 220 cycles. Recently, Zhou and co-workers successfully fabricated a free-standing Sb@carbon nanofiber (Sb@CNF) electrode using an electrospinning method, which exhibited a long lifespan of 2000 cycles.^20^ However, the majority of K-ion anodes are typically prepared via binder-containing slurry processes, which reduces the energy density.^2^ On the other hand, the fabrication of binder-free electrodes is often too complicated to reproduce at a large scale. To overcome these issues, innovative strategies that employ facile methods to produce high-stability Sb anodes for PIBs are desired. Electrophoretic deposition (EPD) is a facile electrode fabrication route without the requirement for binder additives, wherein charged materials travel to and get deposited onto the oppositely charged electrode in the presence of an applied electric field.^21,22^ EPD offers several advantages, including a high deposition rate, suitability for various materials, and the capability to create a uniform, porous, and firmly adherent film on substrates.^21,23,24^ A small number of publications have reported Sb-based anodes formed via EPD for LIBs^23,25−27^ and also CNT-based anode composite materials for PIBs^28−30^ with the electrodes delivering high specific capacity and long-term stability. However, the electrochemical performance of Sb/CNT composite anodes prepared via EPD for PIBs has not been reported in the literature.

In this work, binder-free Sb/CNT nanocomposite anodes with Sb NPs dispersed in MWCNT networks were fabricated by using EPD. The binder-free Sb/CNT electrodes demonstrated outstanding performance in comparison to the binder-free Sb electrodes. The optimized Sb/CNT nanocomposite electrodes exhibited a stable reversible capacity of 341.03 mAh g^–1^ over 300 cycles at 0.2C, while the Sb electrode decayed rapidly after 20 cycles at the same rate. A good rate capability of 183.24 mAh g^–1^ at high rates of 5C was achieved for the optimized Sb/CNT nanocomposite, in contrast to the Sb electrode, which was not able to cycle at this rate. These findings demonstrate that the use of CNT and EPD could enable the upscaling of high-capacity Sb-based anodes for PIBs and other energy storage systems, providing a roadmap for fabricating binder-free alloying metal/carbonaceous composite materials.

## 2 Experimental Section

### 2.1 Chemicals

All of the nanoparticle synthesis was performed using the Schlenk line apparatus to maintain inert conditions. Isopropanol (IPA, ≥99.95%) was purchased from Lennox, Ireland. Antimony trichloride (SbCl_3_, ≥99.95%), sodium borohydride (NaBH_4_, 99.99%), H_2_SO_4_ (98%), MWCNT (>95%), *N*-methyl-2-pyrrolidone (NMP, 99.5%), and Ni(NO_3_)_2_·6H_2_O were bought from Sigma-Aldrich. Battery-grade Cu foil (9 μm thick) was purchased from Pi-Kem. Battery-grade 4 M potassium bis(fluorosulfonyl)imide (KFSI) in 1,2-dimethoxyethane (DME) was purchased from Dodochem, China.

### 2.2 Synthesis

Sb NPs were synthesized via a modified synthesis procedure reported elsewhere.^31^ NaBH_4_ (6 mmol, 0.2296 g) and *N*-methyl-2-pyrrolidone (NMP, 6 mL) were contained and stirred in a three-necked flask, and the solution was evacuated at room temperature for 30 min to remove oxygen and moisture. In parallel, SbCl_3_ (1.5 mmol, 0.3421 g) in NMP (1.2 mL) was prepared inside a glove box with stirring. Next, the solution in the three-neck flask was heated to 60 °C under an Ar atmosphere, and SbCl_3_ in NMP was quickly injected into the flask. The reaction solution quickly turned black, and a water-ice bath was used to cool it down immediately. Sb NPs were removed from the solution by centrifugation (5000 rpm, 5 min) and then rinsed three times with deionized water (30 mL) in order to remove unreacted NaBH_4_ and NaCl side product. Finally, the Sb NPs were dried at room temperature in a vacuum oven overnight.

### 2.3 CNT Treatments

Multiwalled carbon nanotubes (MWCNTs) were added into a solution composed of H_2_SO_4_ and HNO_3_ in a 1:2 ratio, with a concentration of 15 mg mL^–1^. The subsequent mixture was simultaneously refluxed and stirred at 90 °C for 40 min, followed by four washings with DI water and ethanol. Finally, the product was dried under a vacuum at 80 °C overnight before further use.

### 2.4 Electrophoretic Deposition

**Figure 1:**
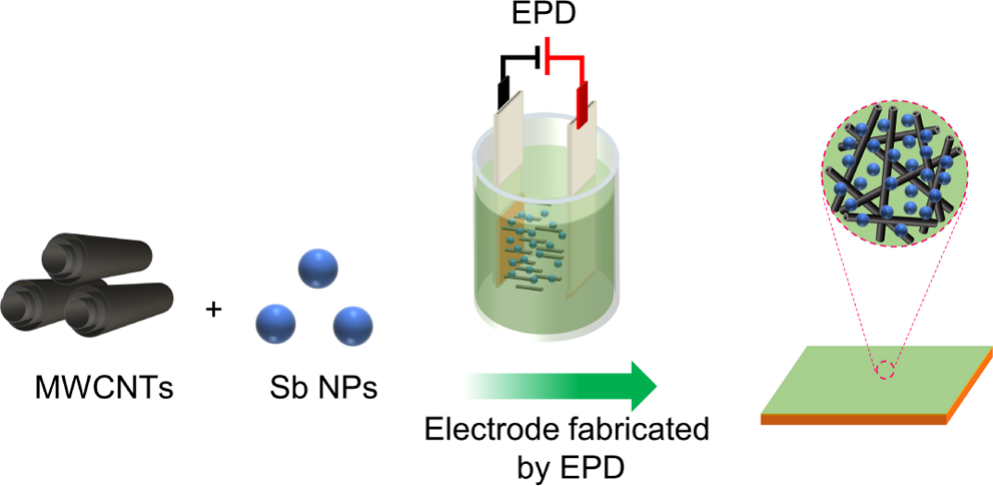
Schematic illustration of binder-free Sb/CNT electrode fabrication using electrophoretic deposition (EPD).

The Sb NP/MWCNT bath was prepared by dispersing Sb NPs and MWCNTs with different mass ratios in IPA with the addition of Ni(NO_3_)_2_·6H_2_O (0.1 mg mL^–1^) with sonication. The MWCNT concentration is fixed at 0.2 mg mL^–1^, and the Sb NP concentration varies depending on the Sb NPs: MWCNT mass ratio. The binder-free Sb/CNT nanocomposite films for PIB electrodes are prepared by immersing Cu foils held at a distance of 2 cm in a Sb NP/MWCNT bath. Subsequently, EPD was conducted in the presence of a direct current (DC) voltage of 300 V (Power supply: TECHNIX SR-5-F-300) for 100 s (as depicted in Figure 1). The Sb NP bath (0.2 mg mL^–1^) and MWCNT bath (0.2 mg mL^–1^) were also prepared to achieve the Sb NP film and the MWCNT film on copper foil, respectively. All of the deposition occurred at the negative electrode. All battery electrodes were dried in a vacuum oven at 80 °C for 12 h with an active material loading of 0.7–0.8 mg cm^–2^. The Sb/CNT nanocomposite electrodes achieved with Sb NPs/MWCNT mass ratios of 1: 1, 2: 1, and 3: 1 were named Sb/CNT-1, Sb/CNT-2, and Sb/CNT-3, respectively. In addition, Sb/CNT electrodes with a higher loading of ∼1.6 mg cm^–2^ were made by varying the total immersion time (200 s) of copper foils in the Sb NP/MWCNT bath.

### 2.5 Characterization

X-ray diffraction (XRD) characterization was investigated using a PANalytical Empyrean instrument with Cu Kα radiation. Raman spectroscopy was carried out on a Horiba Labraman 300 spectrometer system equipped with a 532 nm laser. Scanning electron microscopy (SEM) analysis was carried out using an FEI Helios G4 CX, and transmission electron microscopy (TEM) was conducted using a 200 kV JEOL JEM-2011F microscope equipped with a Gatan camera. The TEM samples were dispersed in IPA under sonication for 15 min, and 10 μL of solution was drop-casted on lacey carbon-coated 200 mesh nickel grids. TEM grids were dried overnight under a vacuum at room temperature before the TEM measurements. X-ray photoelectron spectroscopy (XPS) was conducted on a Kratos AXIS ULTRA spectrometer using a monochromatized Al Kα X-ray gun. Thermogravimetric analysis (TGA) was performed using a TG-DSC analyzer (PerkinElmer TGA 4000) in the air from room temperature to 800 °C (heating rate: 10 °C min^–1^). Postmortem analysis of the electrodes was carried out using SEM and TEM. The cycled cells were disassembled, and then the electrodes were washed in 1,2-dimethoxyethane (DME) and allowed to dry naturally in the glove box overnight before SEM and TEM analyses.

### 2.6 Electrochemical Characterization

An active electrode (12 mm diameter), K metal (99.95%) as a counter electrode, and a glass fiber (GF/D, Whatman) separator comprised the standard half-cell (CR 2032) type, which were assembled in an argon-filled glove box (Vigor). The electrolyte used was 4 M KFSI dissolved in DME. Galvanostatic cycling was carried out using a Neware battery cycler instrument in a potential range of 0.01–1.5 V. The current densities were calculated using the theoretical capacities of the active materials and the total mass loadings of the electrode. All specific capacities in this study were calculated based on the total mass loadings of the electrode. Electrochemical impedance spectroscopy (EIS) was carried out using a BioLogic potentiostat within the 10 kHz to 0.1 Hz frequency range. Cyclic voltammetry (CV) was performed using a BioLogic potentiostat at various scan rates of 0.1–0.9 mV s^–1^. The GITT measurement during the first discharge/charge cycle was performed using a Neware battery cycler instrument at C/5.

## 3 Results and Discussion

**Figure 2:**
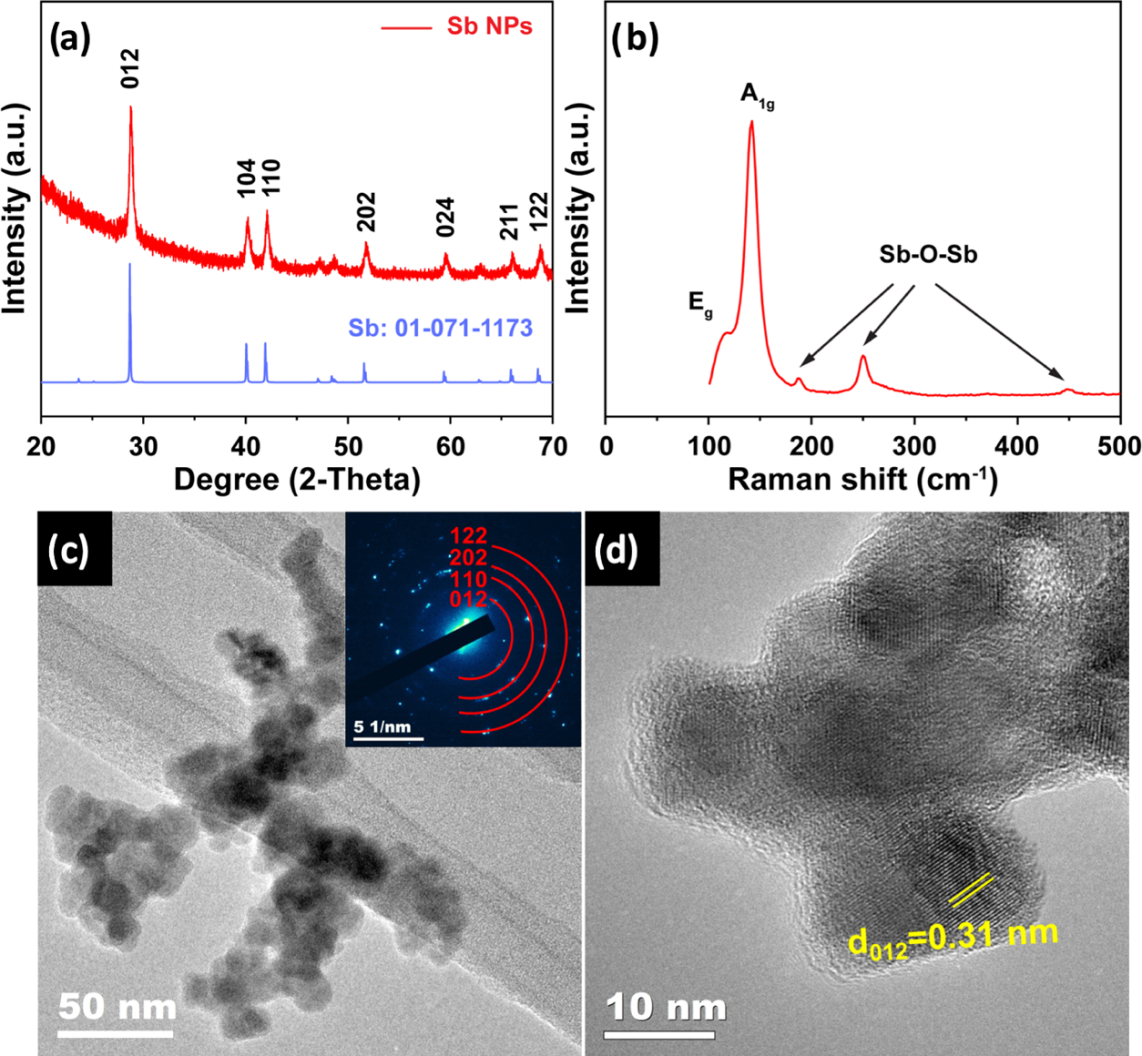
Characterization of the as-prepared Sb NPs: (a) XRD pattern, (b) Raman, (c) TEM image (with the SAED pattern inset), and (d) HR-TEM image.

Figure 2a shows the XRD analysis of Sb NPs achieved after colloidal synthesis. All of the reflections were indexed to the trigonal crystal phase of Sb (JCPDS no. 01-071-1173). No impurity phases were detected, suggesting complete conversion of SbCl_3_ into a Sb metal using the NaBH_4_ reducing agent. The structural characteristics of Sb NPs were also evaluated by Raman spectroscopy (Figure 2b). Raman spectra of Sb NPs showed peaks around 110 and 145 cm^–1^, typically for Sb E_g_ and Sb A_1g_, respectively.^25,32−34^ Weak signals at ∼188 cm^–1^, 249, and 450 cm^–1^ correspond to Sb–O–Sb modes (Sb_2_O_3_ vibrations),^35,36^ suggesting that the synthesized Sb NPs may slightly oxidize owing to their high surface area. In addition, XPS characterization of the Sb NPs was carried out to determine the bonding nature of Sb and O, as shown in Figure S1. Figure S1a shows a wide-scan XPS survey of Sb NPs, confirming the presence of Sb and O elements. The XPS spectra of O 1s and Sb 3d were fitted into eight peaks, observed at 540.9, 539.4, 537, 534.5, 533.1, 531.4, 530, and 527.9 eV (Figure S1b). Among these, two peaks appeared at 534.5 and 533.1 eV arising from the O 1s core-level spectra,^37−39^ while the other peaks can be attributed to the XPS spectra of Sb 3d. The peaks at 540.9 and 531.4 eV can be assigned to Sb^5+^ (Sb_2_O_5_), while the peaks at 539.4 and 530 eV can be assigned to Sb^3+^ (Sb_2_O_3_). The XPS results corroborate with Raman results, confirming the partial oxidation of Sb NPs. The two remaining peaks detected at 537 and 527.9 eV are attributed to Sb^0^ (Sb metal).^37−40^ The TEM image reveals the nanoparticle morphology of the as-prepared Sb, as shown in Figure 2c. The crystal phase of Sb NPs was verified by selected-area electron diffraction (SAED) (Figure 2c inset) taken from an area that includes multiple NPs, showing the distinct diffraction rings of Sb. These results matched well with the XRD results. The HR-TEM revealed the morphology of Sb NPs (Figure 2d), displaying (012) lattice fringes of Sb (*d*-spacing: 0.31 nm). The Sb NPs have an average diameter of 12.04 ± 5.94 nm (Figure S2), as analyzed from TEM images.

The Sb/CNT electrodes were fabricated via EPD to assemble the Sb/CNT on copper foil, as depicted in Figure 1. To make a comparison, pure Sb electrodes and CNT electrodes were prepared separately using the same deposition condition as the Sb/CNT electrodes. Figure S3 displays the morphology of the binder-free electrodes prepared via EPD. A high level of uniformity in the distribution of Sb NPs, CNTs, and the combination of Sb NPs with CNTs assembled onto the copper foil was observed in Figures S3a,d,g, respectively. The higher magnification SEM image (Figure S3b) and cross-sectional image (Figure S3c) of Sb NPs reveal that the Sb NPs appear to be agglomerated after EPD. However, with the use of CNTs in the fabrication of Sb/CNT electrodes, Sb NPs were distributed uniformly within the CNT buffer layer, which can alleviate the volume expansion of Sb NPs during cycling (Figure S3h,i). The TEM images of Sb/CNT (Figure S4) further confirm that the Sb NPs are distributed well onto and within the CNT networks. According to the N_2_ adsorption–desorption isotherms of CNT, Sb, and Sb/CNT samples (Figure S5), the Sb/CNT exhibits a higher specific surface area of 46.4 m^2^ g^–1^ compared to the Sb sample (28.9 m^2^ g^–1^) due to the incorporation of Sb NPs within the CNT networks. This facilitates a high contact area between the active material and the electrolyte in the Sb/CNT sample.^41^

The amount of Sb NPs deposited on the Sb/CNT electrodes could be varied by changing the Sb: CNT mass ratio. However, the mass loadings of Sb NPs and CNTs on the Sb/CNT electrodes might be different from those on the as-prepared Sb: CNT mass ratio. TGA analysis of the CNT in air (Figure S6a) was carried out to determine the background combustion reaction of the CNT, and then, TGA analysis of the Sb/CNT samples was examined to calculate the quantitative composition (Figure S6b–d). Following TGA analysis, the Sb content was calculated to be 55.4, 63.5, and 67.9 wt %, while the carbon content was found to be 44.6, 36.5, and 32.1% for Sb/CNT-1, Sb/CNT-2, and Sb/CNT-3, respectively (Table S1).

**Figure 3:**
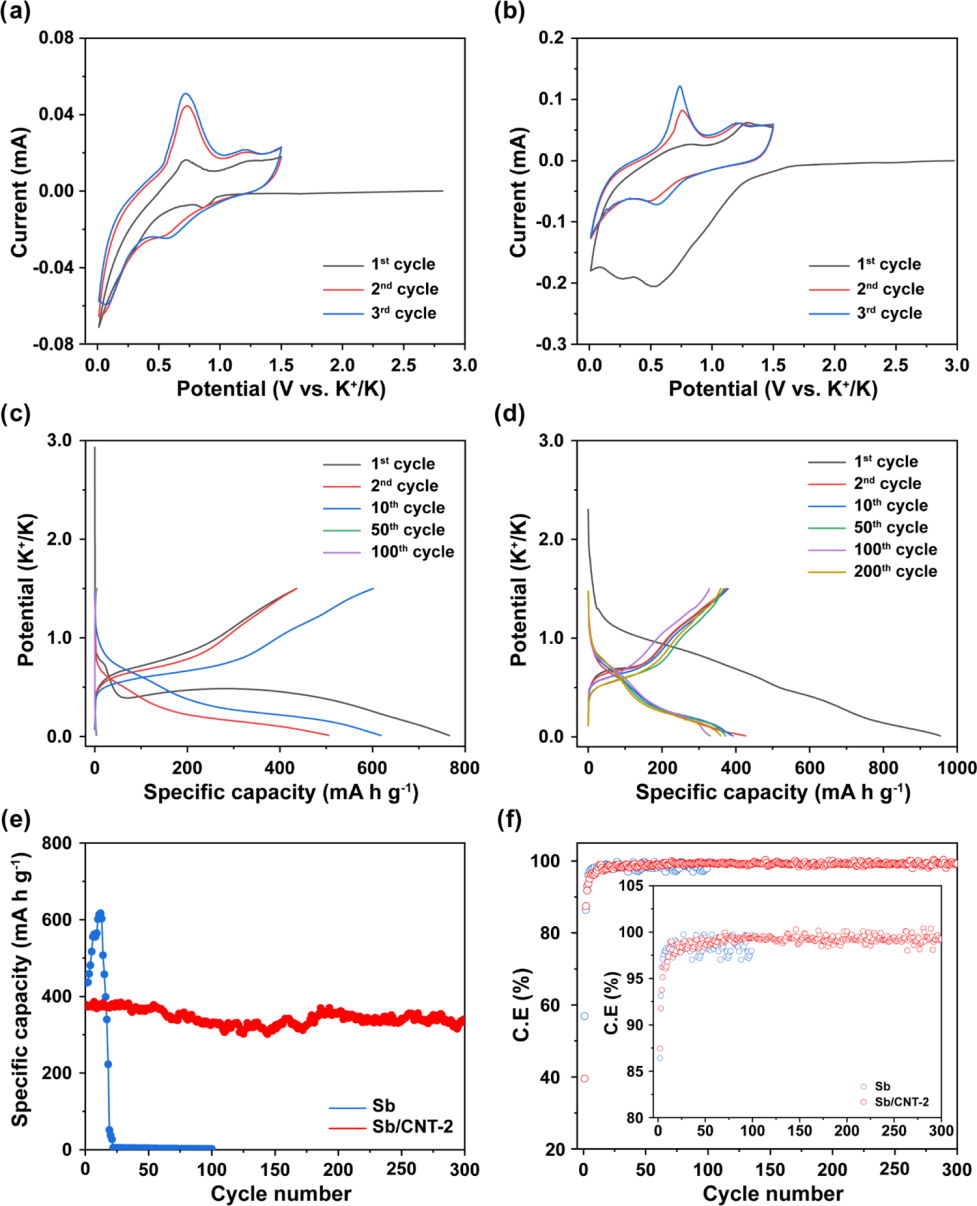
CV curves of (a) Sb and (b) Sb/CNT-2 at a scan rate of 0.1 mV s^–1^. Constant current charge–discharge curves of (c) Sb electrode and (d) Sb/CNT-2 electrode, and comparison of (e) cyclic performance of Sb electrode and Sb/CNT-2 electrode and (f) corresponding Coulombic efficiencies at C/5 (the inset figure shows from the second cycle).

The electrochemical performance of the Sb/CNT composite anodes was investigated in half-cells and compared with that of reference Sb anodes (Sb film deposited on the Cu foil). Figure 3a,b represents the cyclic voltammograms (CV) of the Sb and Sb/CNT-2 electrodes in a potential range of 0.01–1.5 V vs K^+^/K, measured at a scan rate of 0.1 mV s^–1^, respectively. For the Sb-containing electrode, a first small peak appeared at around 0.9 V, predominantly due to electrolyte degradation and solid electrolyte interphase (SEI) formation during the first discharge.^2,12,42^ Afterward, the cathodic current significantly decreased from 0.75 to 0.01 V vs K^+^/K, corresponding to the alloying of K with Sb to form the amorphous-intermediated K_*x*_Sb and then the K_3_Sb alloy phase.^12,14,42,43^ In the subsequent cycles, the reduction peaks at ∼0.57 V and below 0.2 V can be ascribed to the formation of the K_*x*_Sb and K_3_Sb phases, respectively. During the first anodic scan, two broad peaks during oxidation seen at ∼0.75 and 1.20 V may be ascribed to the dealloying reaction of K_3_Sb and the formation of Sb, respectively.^2,14,16,43^ Similarly, the Sb/CNT-2 electrode exhibits the same K-storage behavior as the Sb sample, but the initial cathodic scan was slightly different. Specifically, the cathodic current starts to decrease around 1.5 V, which can be linked to the K^+^ reaction with functional groups present on the CNT surface.^44,45^ This reaction can be seen obviously in the CV of the pure CNT sample (Figure S8a). Then, the formation of the SEI layer and the alloying of K with Sb occur similarly to those in the Sb sample. The redox peaks in the sequential cycles of the Sb sample and Sb/CNT-2 sample display consistent positions and tend to overlap, demonstrating stable SEI formation on the electrode with high reversibility of the alloying/dealloying reactions.^2,14,16,43^ Analogous CV characteristics were identified in the other Sb/CNT electrodes (Figure S9a,b).

Figure 3c,d displays the selected galvanostatic charge/discharge (GCD) profiles of the two samples (Sb and Sb/CNT-2, respectively) at C/5 (∼132 mA g^–1^). The GCD profiles match well with the CV data, showing the same reversible potassiation/depotassiation reactions. As shown in Figure 3c, the first dis(charge) capacities are 766.08 and 436.19 mA h g^–1^ for the Sb electrode with an initial Coulombic efficiency (CE) of 56.9%. The irreversible capacity of Sb is primarily ascribed to the formation of the SEI layer present during the first discharge cycle.^1,46^ In contrast, the Sb/CNT-2 electrode delivered discharge and charge capacities of 954.09 and 377.50 mA h g^–1^, respectively. The CE of the Sb/CNT-2 electrode was 39.5%, which is much lower than that of the Sb. The high irreversible capacity of the Sb/CNT-2 electrode can be attributed to the high surface area of CNTs, which consumes K irreversibly. The CNT electrode exhibits a CE of just 16.4% in the first cycle (Figure S8b). Two plateaus during the discharge process of the Sb electrode at around 0.8 V and below 0.5 V vs K^+^/K can be assigned to the formation of SEI and potassiation of Sb, respectively. In the first charge, two charge potential plateaus located around 0.75 and 1.2 V represent the dealloying process. In contrast, Sb/CNT-2 exhibits a gradually decreasing plateau in the discharge potential when the discharge potential is below 1.5 V vs K^+^/K (Figure 3d). This plateau is attributed to the reaction of functional groups on CNT, the formation of the SEI film, and the potassiation of Sb, corresponding to a significant irreversible capacity during the first cycle. This phenomenon can be seen in all Sb/CNT composite electrodes (Figure S9c,d). In the next step, Sb/CNT-2 shows similar charge potential plateaus at 0.75 and 1.2 V as the Sb electrode. The following GCD profiles of both samples display a consistent potential profile behavior. Fortunately, the Sb/CNT-2 electrode (Figure 3d) and the Sb/CNT nanocomposites (Figure S9c,d) exhibited the almost overlapped profile of the voltage-specific capacity curves from the second to the 200th cycle, suggesting good capacity retention, whereas pristine Sb electrode did not display the similar behavior after 20 cycles.

Figure 3e further illustrates the superior cyclability of the Sb/CNT-2 as compared to the pristine Sb sample. Sb/CNT-2 exhibited a high initial reversible capacity of 377.50 mA h g^–1^ at 0.2C, while the specific capacity of the pure Sb anode was 436.19 mA h g^–1^. The specific capacity of the Sb anode increased to 616.93 mA h g^–1^ after 12 cycles due to the activation process, but it decayed severely in the subsequent cycles. The Sb anode could not function effectively as an anode for K-storage after ∼20 cycles. In contrast, Sb/CNT-2 still maintained a stable charge capacity of 341.30 mA h g^–1^, which is a capacity retention of 90.41% after 300 cycles. The incorporation of Sb NPs with CNTs, encapsulating individual Sb NPs within the 3D interconnected CNT networks, is believed to enhance the K-storage ability, as it facilitates the participation of each Sb NP in the cycling process. Meanwhile, the other composite electrodes Sb/CNT-1 and Sb/CNT-3 also exhibited comparable reversible capacities to the Sb/CNT-2, as shown in Figure S10a. Besides, the CNT plays a crucial role in improving the stable cycling performance of the Sb/CNT electrodes. Sb/CNT-1 and Sb/CNT-3 achieved a good reversible capacity of 289.54 mA h g^–1^ (94.86% of the initial capacity) and 258.89 mA h g^–1^ (65.38% of the initial capacity) after 300 cycles, respectively (Figure S10a). Sb/CNT-3 shows inferior cyclic stability compared to Sb/CNT-1 and Sb/CNT-2 because the low CNT amount may not be sufficient to avoid Sb NPs’ agglomeration over a long-term cycling. However, increasing the CNT content in Sb/CNT nanocomposites can decrease the initial CEs of the Sb/CNT electrodes because a significant amount of K^+^ reservoir is consumed for SEI layer formation on the surface of CNT (Figures 3f and S10b). Besides, a high CNT content can lead to the low total specific capacity of Sb/CNT electrodes because the CNT electrode delivered a reversible capacity of <50 mA h g^–1^ (Figure S8b), which is much lower than the specific capacity of Sb. Consequently, with the increase in the CNT content from 32.1% (Sb/CNT-3) to 44.6% (Sb/CNT-1), the specific capacity decreased by approximately 90.72 mA h g^–1^. Therefore, among the prepared electrodes, the Sb/CNT-2 electrode was utilized for further investigation due to its appropriate reversible capacity, with decent Coulombic efficiency and cycling stability.

**Figure 4:**
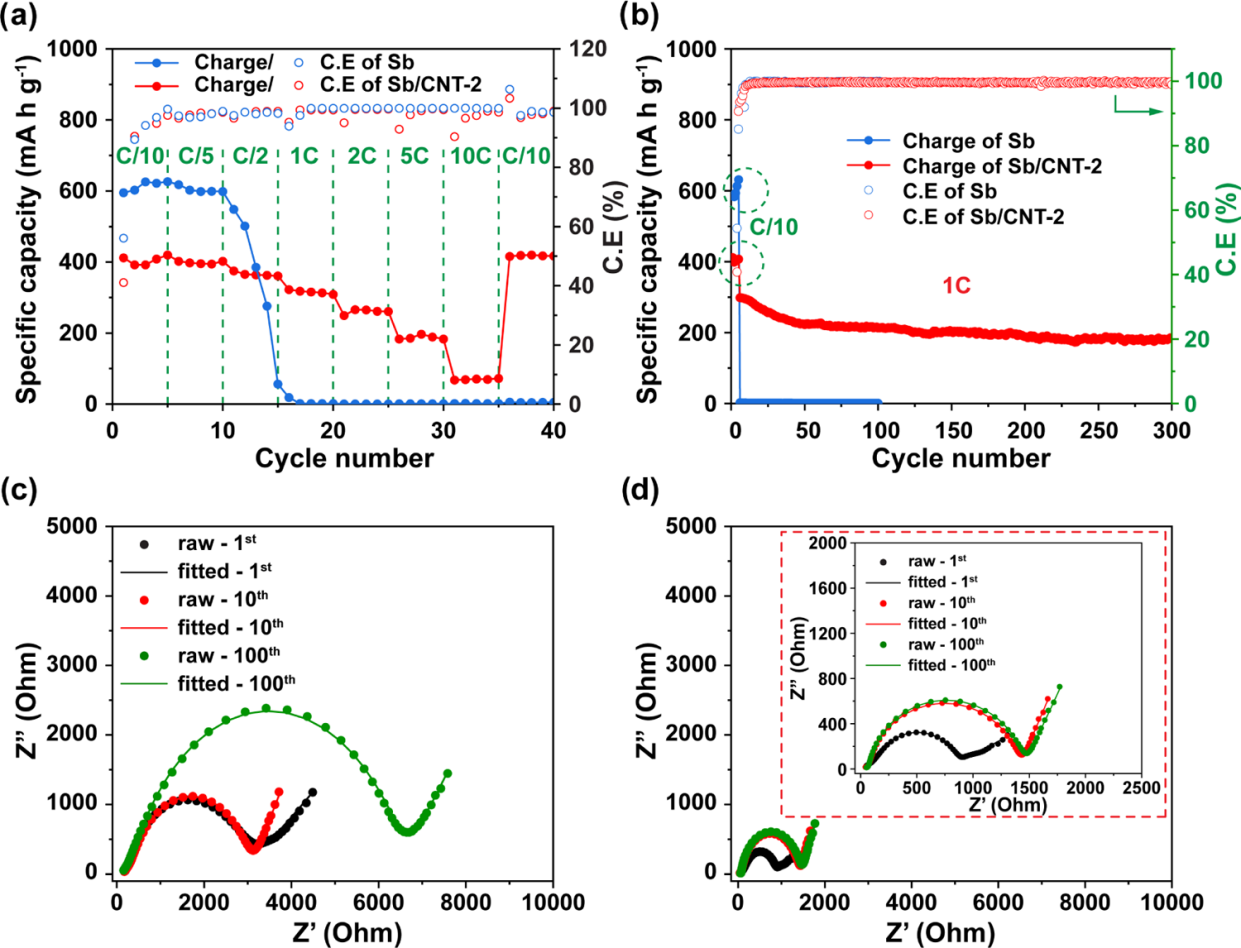
(a) Rate performances of Sb and Sb/CNT-2. (b) Cyclic and corresponding Coulombic efficiency profiles of Sb and Sb/CNT-2 electrodes at a rate of 1C with first 5 cycles at C/10 for activation. Nyquist plots of (c) Sb and (d) Sb/CNT-2.

Rate capability testing was investigated, ranging from C/10 to 10C (1C ∼ 660 mA g^–1^) for five cycles each (Figure 4a). At rates of C/10 and C/5, the Sb/CNT-2 electrode delivered an average reversible capacity of 419.80 and 402.20 mA h g^–1^, while the specific capacities of the Sb electrode were 626.67 and 598.95 mA h g^–1^. When the C-rate increased from 0.5C to 10C, the cycling performance of the Sb electrode declined significantly and was not able to deliver a high capacity when the current rate returned to C/10. In contrast, the Sb/CNT-2 electrode exhibited superior rate capacity, exhibiting average reversible capacities of 361.01, 308.99, 260.87, 183.24, and 72.50 mA h g^–1^ at rates of C/2, 1C, 2C, 5C, and 10C, respectively. When the current density returned to the initial value of 0.1C, a high capacity of 417.08 mA h g^–1^ was retained, exhibiting a good capacity retention of 99.3% after cycling at high C-rates. The rate capability of the Sb/CNT-2 electrode outperforms that of the Sb electrode for PIBs, which is mainly attributed to the Sb/CNT-2 nanocomposite structure, as well as due to the CNT buffer layer, mitigating volume changes during cycling, consecutively ensuring good electronic transport within the electrode, and further improving the electrode performance at high current densities. Moreover, the long-term resilience of the Sb/CNT-2 electrode was investigated at high C- rates (1C ∼ 660 mA g^–1^) for 300 cycles (Figure 4b). A rate of C/10 was applied for the first five cycles to activate the electrode, and the following cycles were at a rate of 1C. After initial activation at the modest current rate of C/10, Sb/CNT-2 exhibited a reversible capacity of 298.69 mA h g^–1^ while the Sb electrode degraded rapidly and did not show any signs of K-storage at 1C. The reversible capacity of Sb/CNT-2 faded quickly to ∼222.89 mA h g^–1^ after 50 cycles but then maintained a stable cycling capacity of 185.69 mAh g^–1^ even after 300 cycles. The rapidly faded capacity can be attributed to the mechanical deterioration and the formation of an unstable SEI in a few initial cycles at a high current rate. To understand the stability of the Sb/CNT-2 nanocomposite electrode, electrochemical impedance spectroscopy (EIS) was measured. The Nyquist plots of the Sb and Sb/CNT-2 electrodes (Figure 4c,d, respectively) were fitted with an equivalent circuit depicted in Figure S11. The fitted EIS data are listed in Table S2. Both materials showed comparable *R*_SEI_ values of 143.8 (Sb electrode) and 166.6 Ohm (Sb/CNT-2) after the first cycling, as shown in Table S2. While *R*_SEI_ of Sb/CNT-2 decreased gradually to 53.26 Ohm at the 100th cycle, *R*_SEI_ of Sb emerged quickly to 467.3 Ohm at the 100th cycle. The reduction of R_SEI_ of Sb/CNT-2 suggests that the SEI layer stability gradually increases during long cycling. In contrast, the unstable SEI layer on the Sb electrode resulted in high SEI resistance. The *R*_s_ and *R*_ct_ of Sb and Sb/CNT-2 increased over a number of cycles. *R*_s_ and *R*_ct_ of Sb were 70.76 and 2272 Ohm after the first depotassiation and then rose to 96.87 (*R*_s_) and 5984 Ohm (*R*_ct_) after 100 cycles, while Sb/CNT-2 showed lower values of *R*_s_ and *R*_ct_, particularly 20.64 and 632.8 Ohm after the first cycle and 22.55 and 1378 Ohm after 100 cycles. This accounted for the greater specific area of CNT matrix incorporating with Sb NPs, which facilitates a favorable charge transfer mechanism by enabling more active K-ion transfer sites and the construction of a good electron transport network on the electrode surface, thereby enhancing the conductivity of the Sb/CNT electrodes. This is deemed to be the main reason why the Sb/CNT nanocomposites could perform well at a high current rate and exhibit stable long-term cycling. The GITT was used to elucidate the enhancement of ionic conductivity of the Sb/CNT-2 electrode compared to the pure Sb. The K^+^ diffusivity in Sb/CNT-2 was higher than that in pure Sb throughout both the charge and discharge operations, as illustrated in Figure S13. The ex situ impedance of the Sb/CNT-2 sample was further measured in order to better represent the electrochemical reaction process of the initial charging and discharging procedures. The EIS of the initial charging and discharging processes at various voltages is displayed in Figure S14. The Nyquist plot of the cell before cycling (open-circuit voltage, OCV) was fitted with the circuit model shown in Figure S15, while other plots at different voltages were fitted with an equivalent circuit depicted in Figure S11. The fitted EIS data are listed in Table S3. The fresh cell shows a high *R*_ct_ of 12595 Ohm, but upon discharge to 1.0 V, it rapidly decreases to 5022 Ohm, and then it gradually alters during the discharge procedure to 0.01 V. *R*_ct_ continues to move downward during the charging procedure. *R*_ct_ has decreased to just 616.6 Ohm when charged to 1.5 V. Regarding *R*_SEI_, its value decreases significantly from 1.0 to 0.8 V while discharging (Table S3). Following that, R_SEI_ continues to reduce. As a result, the Sb/CNT-2 electrode is continuously active during the first cycle, which helps to lessen polarization.^47^

**Figure 5:**
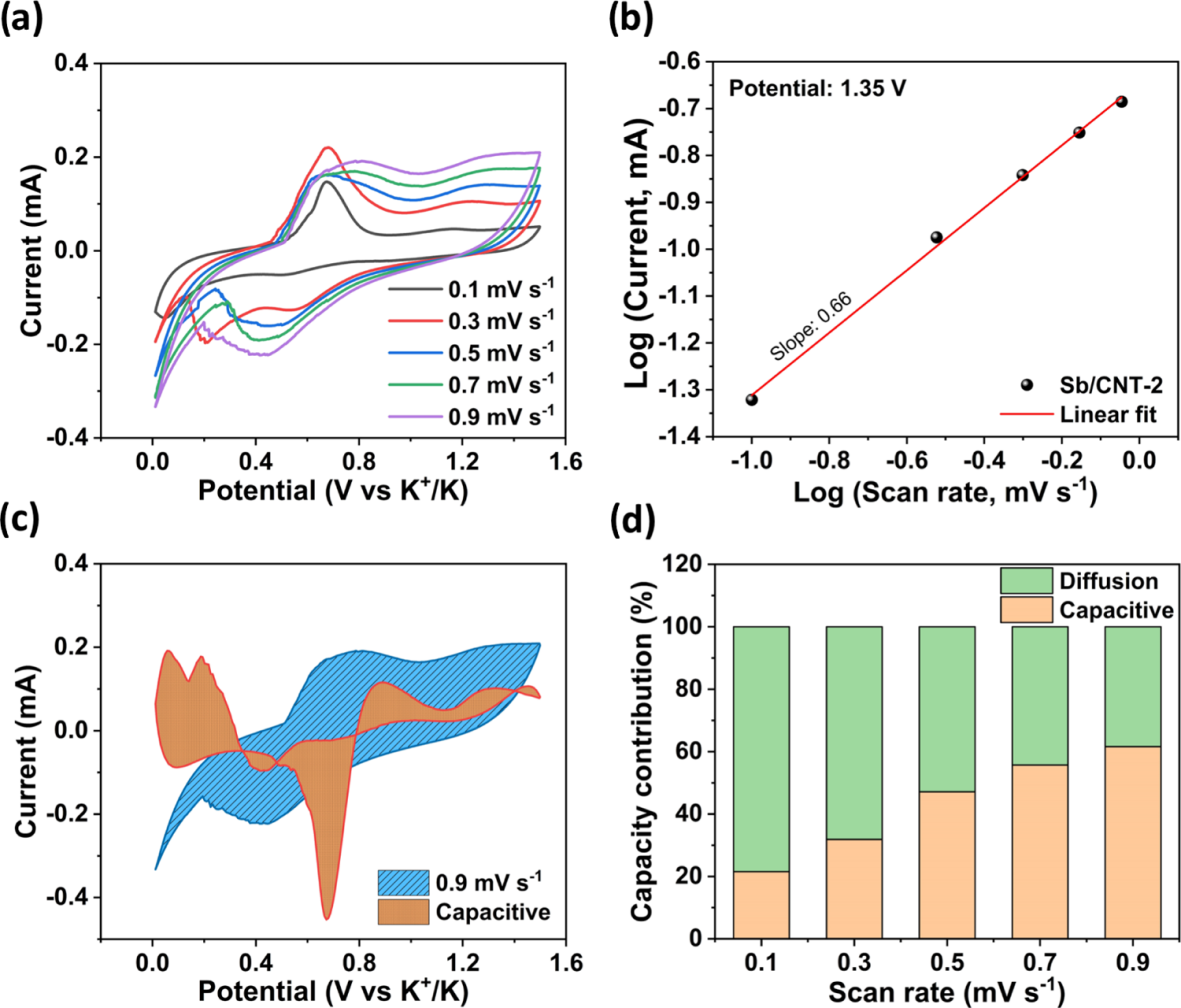
(a) CV cycles of the Sb/CNT-2 electrode at scan rates at various scanning rates. (b) Linear regressions for plots of logarithmic i vs logarithmic v at a cathodic peak potential at 1.35 V. (c) Capacity separation analysis of Sb/CNT-2 at a current of 0.9 mV s^–1^. (d) Capacity contributions of Sb/CNT-2 at various scanning rates.

To further investigate the electrochemical properties of Sb/CNT-2 electrodes, the CV curves were recorded and analyzed at different sweep rates ranging from 0.1 to 0.9 mV s^–1^ (Figure 5a). The relationship between the scan rate (ν) and the current (i) in the CV data can be obtained using the power law *i* = *a*ν^*b*^, where *a* and *b* are the adjustable parameters. A *b* value of 0.5 denotes a slow-driven diffusion process, while a value of 1 represents a fast near-surface Faradaic process.^48^ The b-value for the Sb/CNT-2 anode was estimated to be 0.66 (Figure 5b), suggesting a combination of both slow-driven diffusion and fast near-surface Faradaic process leading to good cycling stability and rate capability. The capacitive and diffusion-controlled contributions of the Sb/CNT-2 electrodes at different scan rates were obtained using the formula, *i* = *k*_1_ν + *k*_2_ν^1/2^, where *k*_1_ν and *k*_2_ν^1/2^ represent the capacitive and diffusion behaviors. Using a representative scan rate of 0.9 mV s^–1^, the Sb-CNT-2 anode presented a capacitive contribution of 62% (Figure 5c). Furthermore, the capacitive contributions for Sb/CNT-2 at 0.1, 0.3, 0.5, and 0.7 mV s^–1^ were estimated to be 22, 32, 47, and 56%, respectively (Figure 5d) from the representative scan rates (Figure S16). The impact of mass loading on the cyclability was carried out on the Sb/CNT-2 electrode, which was two times greater than the previous mass loading of 0.8 mg cm^–2^, as shown in Figure S17. Despite the higher mass loading, the Sb/CNT-2 electrode provided a high reversible capacity of 372.82 mA h g^–1^ in the first cycle, suggesting that the 3D interconnected CNT nanoscale network enables good electrolyte penetration, facilitating the activation of Sb NPs to be activated. The electrode with the higher mass loading showed a reversible capacity of 269.69 mA h g^–1^, equivalent to 72.33% of the initial capacity (0.27% deterioration after each cycle) after 100 cycles. The Sb/CNT-2 electrode with the high mass loading showed respectable cycling stability, not abruptly degrading with cycling as required for its actual use.

**Figure 6:**
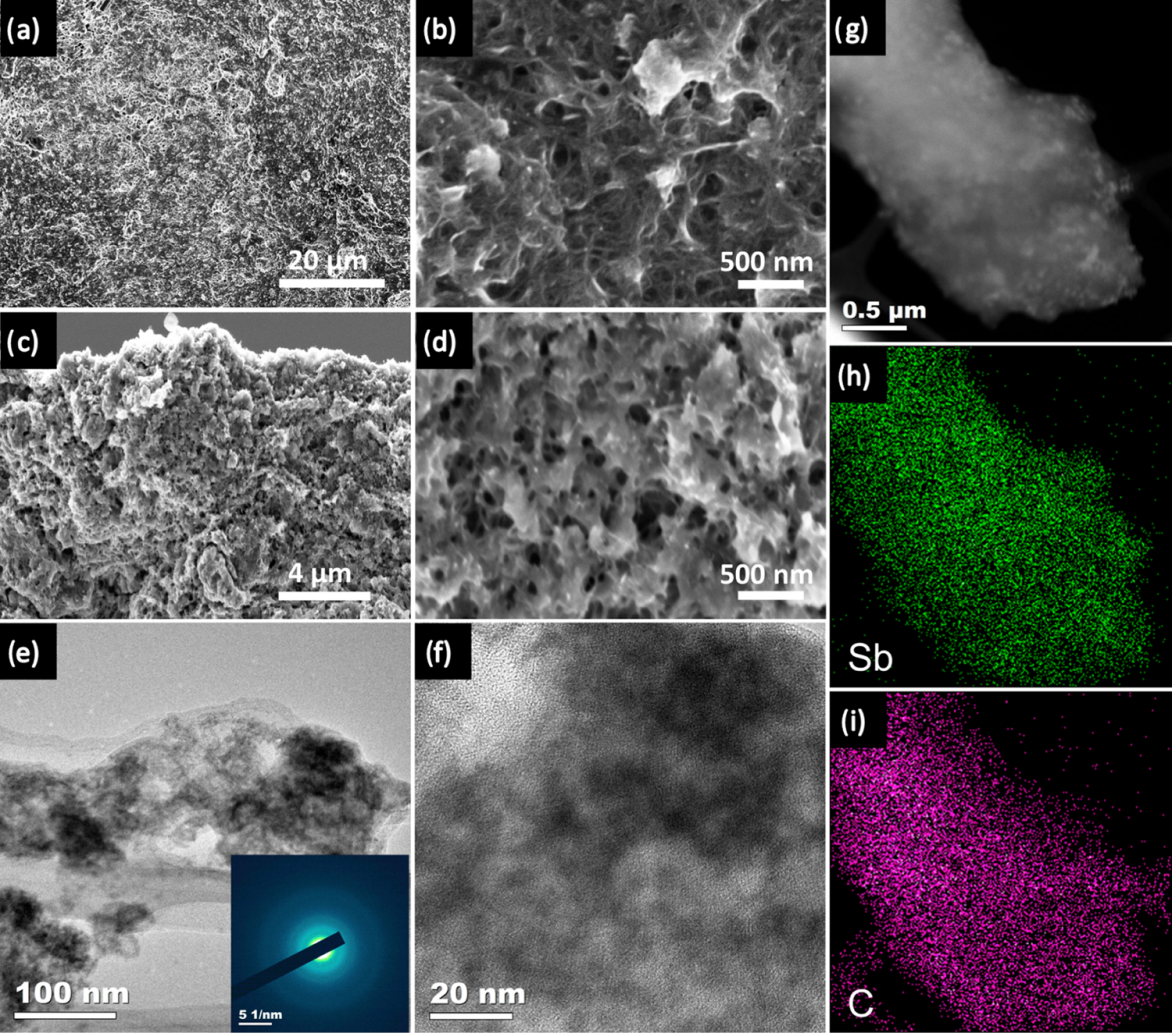
Postmortem characterization of the Sb/CNT-2 after the 100th cycle: (a, b) Top–down SEM images and (c, d) cross-section SEM images, (e) TEM image with the SAED pattern (inset) and (f) HR-TEM image, and (g) STEM with (h, i) elemental mapping images of C and Sb.

To explain the stability of the Sb/CNT-2 electrode, the structural and morphological stabilities of the Sb/CNT-2 after the cycling test were investigated using optical microscopy, SEM, TEM, and EDS mapping. Optical photographs of the Sb and Sb/CNT-2 electrodes (Figure S18) in the charged state after 100 cycles were compared with the untested electrodes. White spots were observed that corresponds to the remaining glass fiber. There was no evidence of leaching in the examined electrode, suggesting the stable binding of the active material with the current collector. The top–down and cross-sectional SEM images reveal morphological changes of the control Sb electrodes (Figure S19), with noticeable cracks and agglomeration on the electrode. In contrast, the nanocomposite structure of Sb/CNT-2 was maintained with a uniform distribution of Sb NPs within the 3D interconnected CNT matrix (Figure 6a,b (top–down) and Figure 6c,d (cross-section)), ensuring that the stress/strain caused by the volume changes during cycling could be tolerated by the 3D interconnected CNT matrix. In addition, the TEM images further confirm the nanocomposite structure of Sb/CNT-2 (Figure 6e,f). The integrity of the Sb NPs was sustained by CNT buffer layers, alleviating the volume expansion of the Sb NPs over the number of cycles. The crystal phase of Sb NPs was converted to the amorphous phase, as evident by the SAED pattern (Figure 6e inset). Furthermore, the uniform distribution of Sb and C was verified by the EDS mapping (Figure 6h, (i)), along with the STEM image (Figure 6g). This demonstrates the good structural stability of the Sb/CNT-2 electrode. The exceptional ability of Sb/CNT-2 to maintain stability and enable extensive cycling can be attributed to the advantageous impact of the interconnected CNT matrix. The interconnected CNT structure serves as a protective layer, offering sufficient spaces to accommodate the expansion of Sb and mitigating the stress/strain induced by the volume exchange during cycling.

## 4 Conclusions

In summary, the direct assembly of Sb/CNT nanocomposite electrodes on the current collector was accomplished using an EPD technique, containing Sb NPs as the active material distributed uniformly in a conductive CNT matrix. The reported method enables large-scale fabrication of binder-free Sb/CNT electrodes due to its simplicity and cost-effective nature of the process, avoiding the use of inactive materials (i.e., binders). The fabricated Sb/CNT nanocomposite structure consists of a unique 3D interconnected CNT matrix anchoring Sb NPs, facilitating electrical contact throughout the electrode while also accommodating the volume changes of Sb NPs during cycling and maintaining the structural integrity of the Sb/CNT nanocomposite electrode. Based on the structural advantages, Sb/CNT outperforms the Sb electrode in terms of cycling stability and rate capability. A comprehensive postmortem analysis of these anode samples using optical imaging, SEM, and TEM revealed more details on the good electrical connectivity, mechanical stability of the electrodes, and cyclic stability of the Sb/CNT-2 electrode fabricated by EPD. The work shows that binderless composite anodes with active materials codepostied with conductive CNTs are an effective approach to PIBs with good cycle stability.

## Supporting Information Available

XPS spectra, SEM, TEM, and TGA results, EDS element mapping results, CV curves, galvanostatic cycling tests, diameter distribution, GITT, EIS, and XRD patterns (PDF)
