# Supplementary material for: Binder-Free Anodes for Potassium-ion Batteries Comprising Antimony Nanoparticles on Carbon Nanotubes Obtained Using Electrophoretic Deposition
Source: ACS Appl Mater Interfaces. 2024 Jul 1;16(27):34809–18. doi: 10.1021/acsami.4c02318 (PMC11247428; doi:10.1021/acsami.4c02318)
Supplement: Supplementary file 1 — am4c02318_si_001.pdf [file am4c02318_si_001.pdf]

Supporting Information

Binder-free Anodes for Potassium-ion Batteries comprising Antimony Nanoparticles on Carbon Nanotubes obtained using Electrophoretic Deposition

*Xuan-Manh Pham, Syed Abdul Ahad, Niraj Nitish Patil, Maria Zubair, Misbah Mushtaq, Hui Gao, Kwadwo Asare Owusu, Tadhg Kennedy, Hugh Geaney, Shalini Singh, Kevin M. Ryan**

Department of Chemical Sciences and Bernal Institute, University of Limerick, Limerick, V94 T9PX, Ireland

E-mail: Kevin.M.Ryan@ul.ie

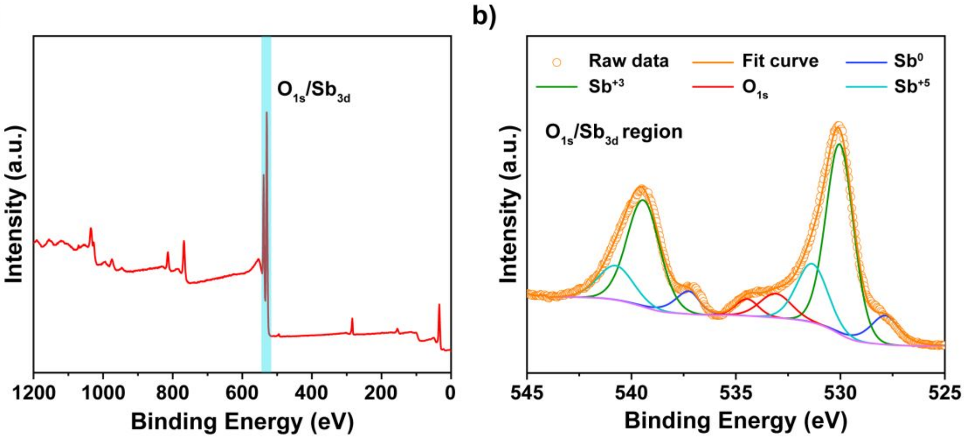

Figure S1. XPS spectra of Sb NPs: (a) wide scan spectrum and core peaks corresponding to (b) O 1s / Sb 3d.

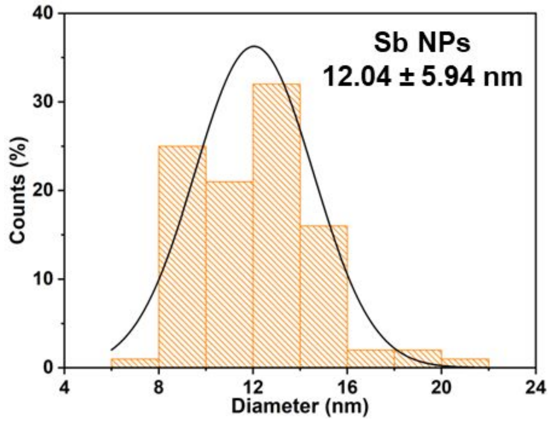

Figure S2. Diameter distribution of Sb NPs.

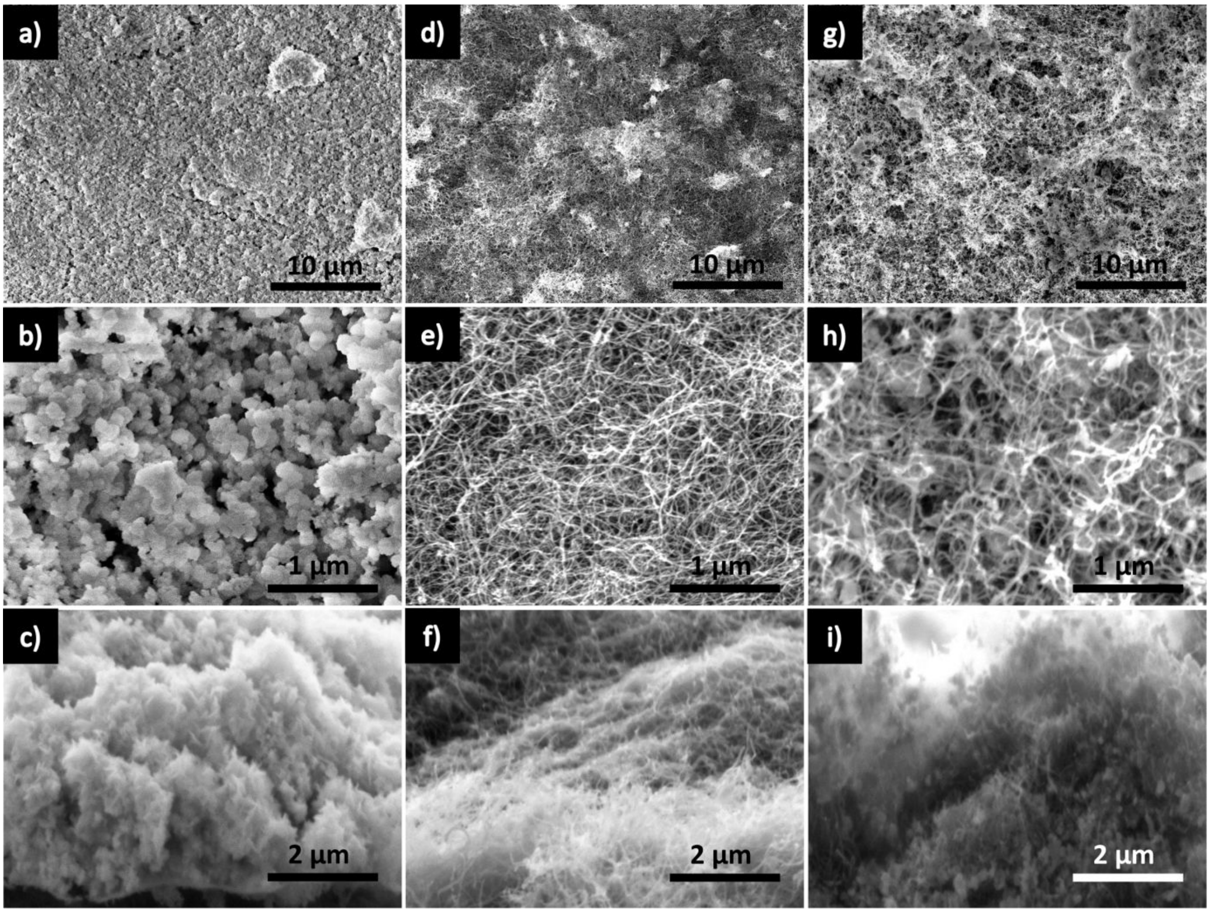

Figure S3. Top-down SEM images of (a, b) Sb NPs, (d, e) CNTs and (g, h) Sb/CNT deposited by EPD. Cross-section SEM images of (c) Sb NPs, (f) CNTs and (i) Sb/CNT deposited by EPD.

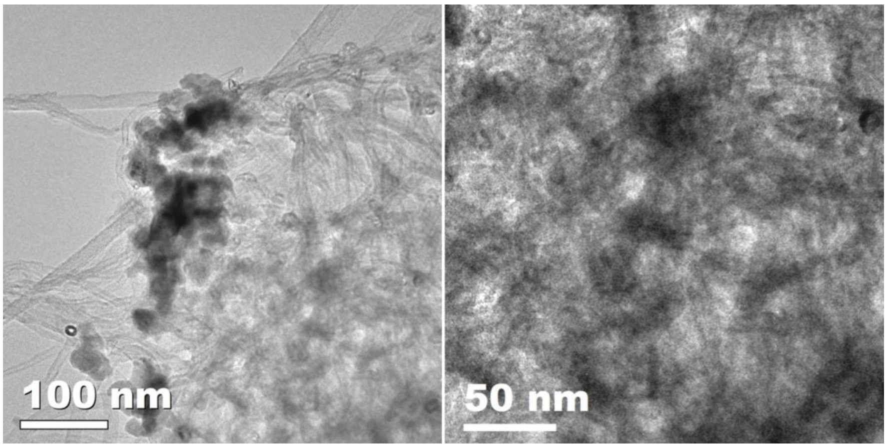

Figure S4. TEM images of the Sb/CNT nanocomposite.

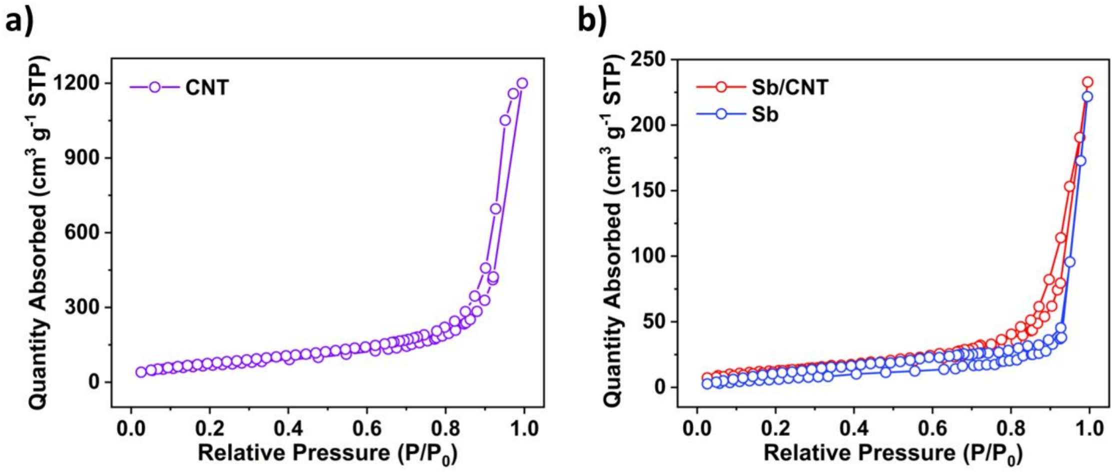

Figure S5. N_2 adsorption-desorption isotherms of (a) CNT and (b) Sb and Sb/CNT.

The Brunauer-Emmett-Teller (BET) surface areas of CNT, Sb and Sb/CNT are 251.1 $m^2\ g^{-1}$ (Figure S5a), 28.9 $m^2\ g^{-1}$ and 46.4 $m^2\ g^{-1}$ (Figure S5b), respectively.

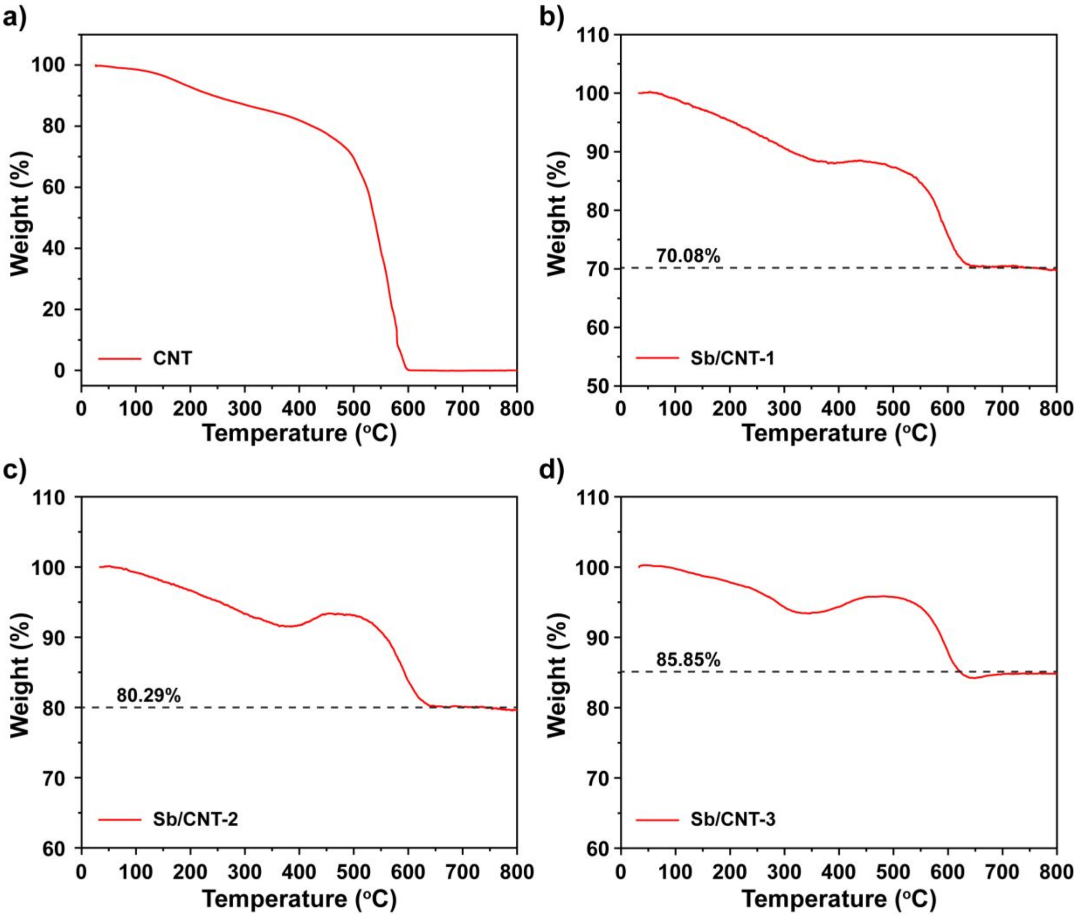

Figure S6. Thermogravimetric (TG) plots of (a) CNT, (b) Sb/CNT-1, (c) Sb/CNT-2, and (d) Sb/CNT-3 up to 800 °C in air.

Figure S6a reveals the weight loss of the CNT sample near 100 °C due to the removal of moisture, then the combustion of oxygen-containing functional groups present in CNT to 500 °C, and finally the burning of the remaining carbon in air observed from ~ 500 °C to 600 °C.[1] It is recognized that all Sb/CNT samples exhibit similar TGA behaviours, including the combustion reaction of CNT and the oxidization process of Sb. Initially, the weight loss observed was due to moisture and oxygen-containing functional groups as described earlier. As the temperature steadily increased, a slight weight gain in the samples observed from ~350 °C to 500 °C was attributed to the partial oxidation of metallic Sb into Sb_2O_3 ($4Sb + 3O_2 \rightarrow 2Sb_2O_3$), as shown in Figure S6b-d. The co-existence of Sb and Sb_2O_3 phases was confirmed by the XRD result of Sb/CNT samples at 500 °C (Figure S7a). After that, the Sb/CNT samples exhibited significant weight loss, corresponding to carbon combustion of CNT ($C + O_2 \rightarrow CO_2$). In the meantime, the further oxidation of Sb_2O_3 ($2Sb_2O_3 + O_2 \rightarrow 2Sb_2O_4$) as well as the residual Sb ($2Sb + 2O_2 \rightarrow Sb_2O_4$) happen together.[2-4] Finally, the weight of the Sb/CNT composite samples remained stable at temperatures above 600 °C. The XRD pattern of the oxidation product of the Sb/CNT composite after the heating process confirming the Sb_2O_4 (SbO_2) phase is presented in Figure S7b. The weight percentage of Sb present in the Sb/CNT composite was calculated as follows.

$$Sb\ wt\% = \frac{Weight(\%)_{at 800^{o} C}}{M_{SbO_2}} \text{ x } M_{Sb}$$

where M_{Sb} and M_{SbO_2} are the molecular weights of Sb and SbO_2 respectively, and the weight(%) at 800 °C is the assumed weight of SbO_2.

Table S1. The quantitative composition of Sb/CNT nanocomposites. Presumably, Sb/CNT samples only consist of Sb and C.

Samples	Weight (%) at 800 °C	Calculated Sb wt%	Calculated CNT wt%
Sb/CNT-1	70.08	55.4	44.6
Sb/CNT-2	80.29	63.5	36.5
Sb/CNT-3	85.85	67.9	32.1

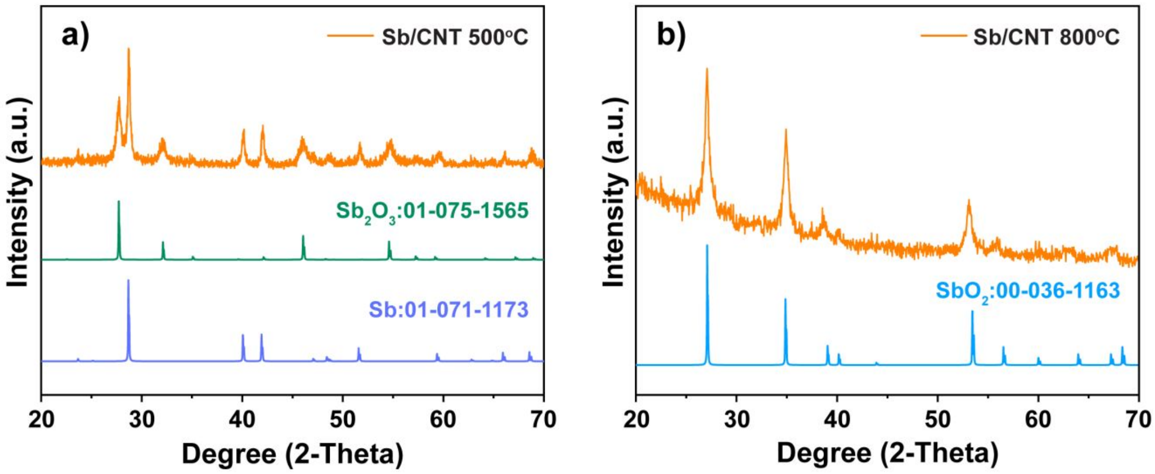

Figure S7. XRD patterns of Sb/CNT composite samples after heating at (a) 500 °C and (b) 800 °C in air.

The XRD pattern of Sb/CNT sample after heating at 500 °C exhibited peaks corresponding to Sb_2O_3 as well as Sb (Figure S7a), confirming the weight gain in the TGA curve of Sb/CNT samples (Figure S6b-d) related to the first-step oxidation of Sb. All the diffraction peaks of the Sb/CNT sample after heating at 800 °C were attributed to the standard diffraction pattern of SbO_2 (Figure S7b).

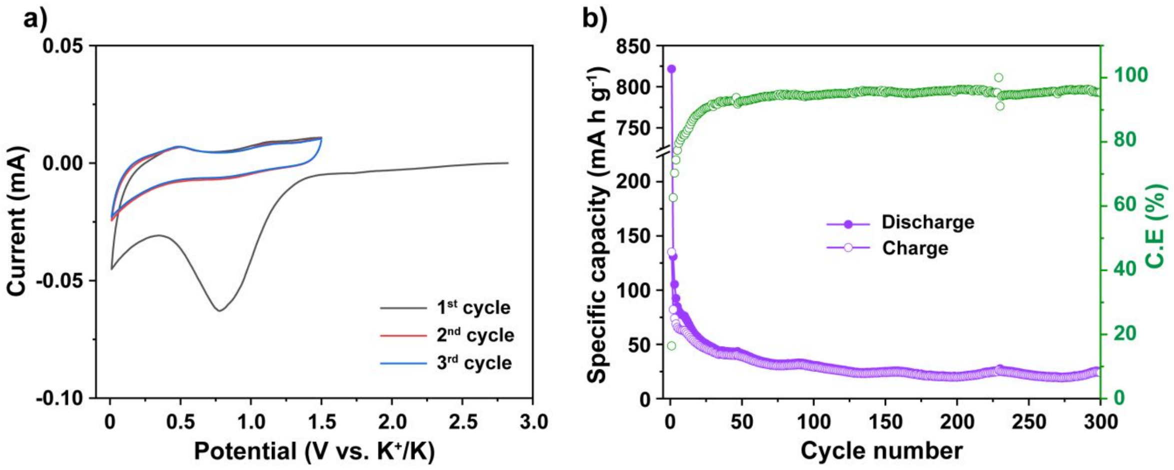

Figure S8. (a) CV of CNT electrode at scan rate 0.1 mV s^{-1} and (b) cycling performance at C/5 of CNT electrode.

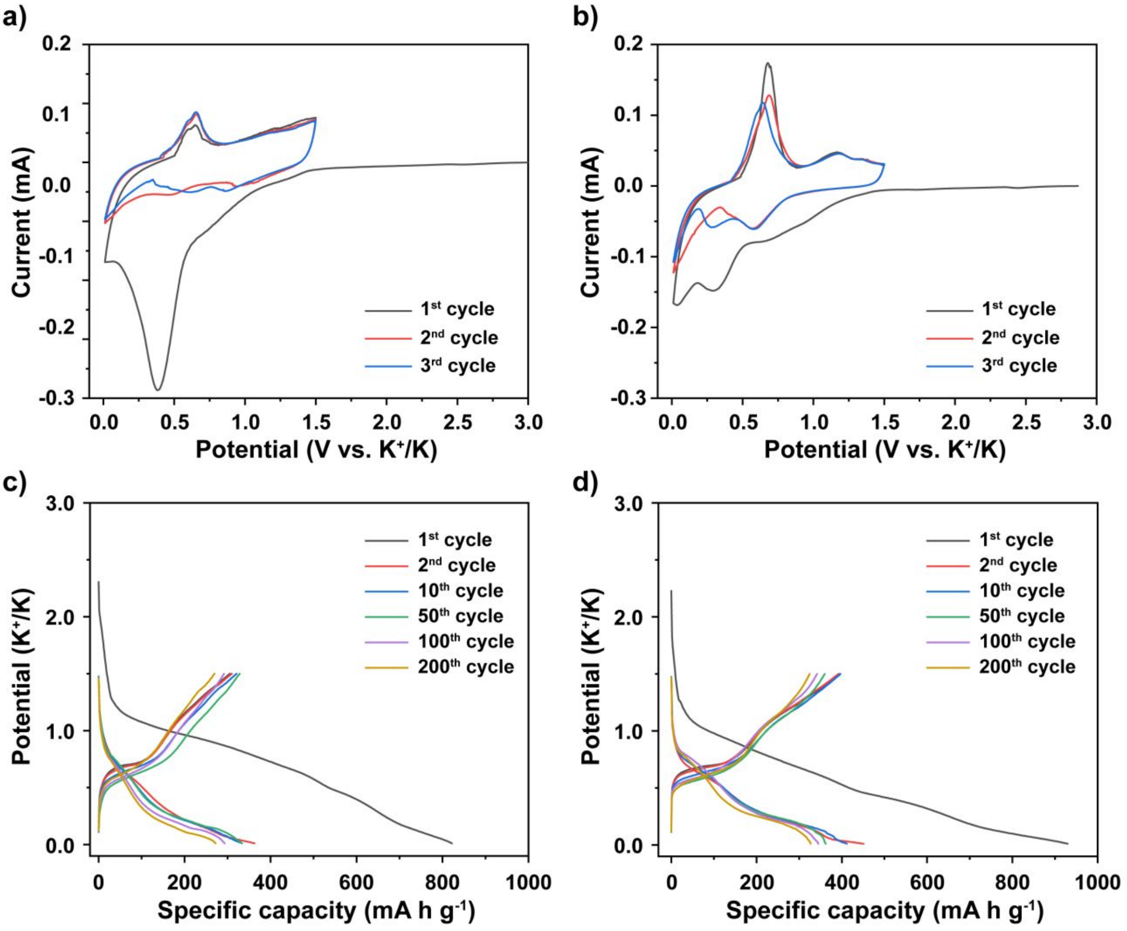

Figure S9. CV of (a) Ge/CNT-1 and (b) Ge-CNT-3 electrodes at scan rate 0.1 mV s^{-1}. Galvanostatic charge-discharge profiles of c) Sb/CNT-1 and d) Sb/CNT-3 electrodes at C/5.

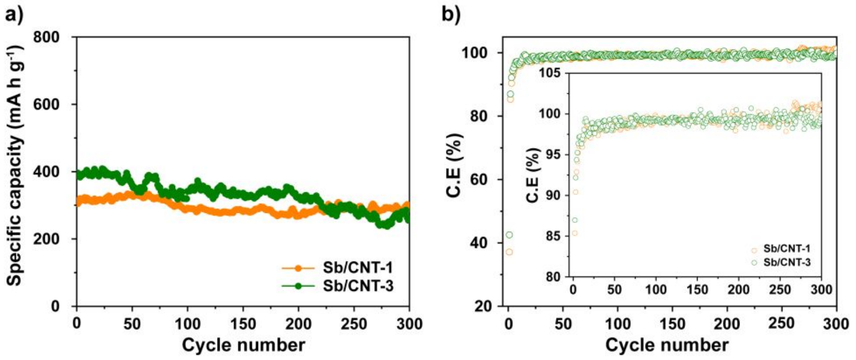

Figure S10. (a) Cyclability with (b) coulombic efficiency of Sb/CNT-1 and Sb/CNT-3 electrodes at C/5 (the inset figure shows from the 2nd cycle).

The Sb/CNT-1 and Sb/CNT-3 electrodes delivered specific capacities of 305.21 and 395.93 mA h g^{-1}, respectively, in the first cycle at 0.2C. Sb/CNT-3 exhibited an initial CE of 42.5% (Figure S8b), higher than the CEs of Sb/CNT-2 (39.5%, Figure 3f) and Sb/CNT-1 (37.1%, Figure S8b) in the first cycle. The effect of CNT content on the CE of Sb/CNT continues in a few first cycles, the CEs of all Sb/CNT electrodes only reached over 96% after 10 cycles and maintained around 98% after 15 cycles whereas the CE of Sb electrode (Figure 3f) was over 97% after 5 cycles and maintained around 98% after that.

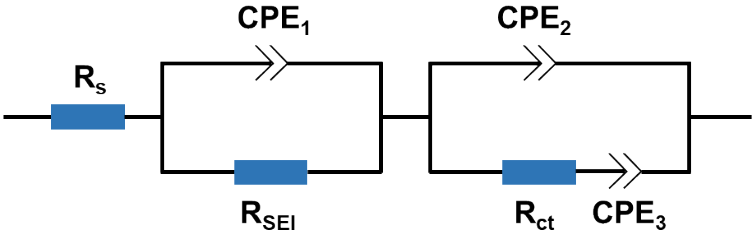

Figure S11. An equivalent circuit used for fitting of EIS data.

The equivalent circuit includes R_s, R_{SEI}, and Rct which indicate the resistance of the electrolyte, the resistance of the SEI layer and the charge transfer resistance, respectively. CPE_1, CPE_2, and CPE_3 denote the capacitances corresponding to the charge-transfer process.

Table S2. Tabulation of R_s, R_{SEI} and R_{ct} value of Sb/CNT-2 and Sb at different cycle no. cycled.

Electrode	Sb/CNT-2			Sb		
Cycle	R_s (Ω)	R_{SEI} (Ω)	R_{ct} (Ω)	R_s (Ω)	R_{SEI} (Ω)	R_{ct} (Ω)
1st	20.64	166.6	632.8	70.76	143.8	2772
10th	21.22	55.38	1339	91.2	250.3	2789
100th	22.55	53.26	1378	96.87	467.3	5984

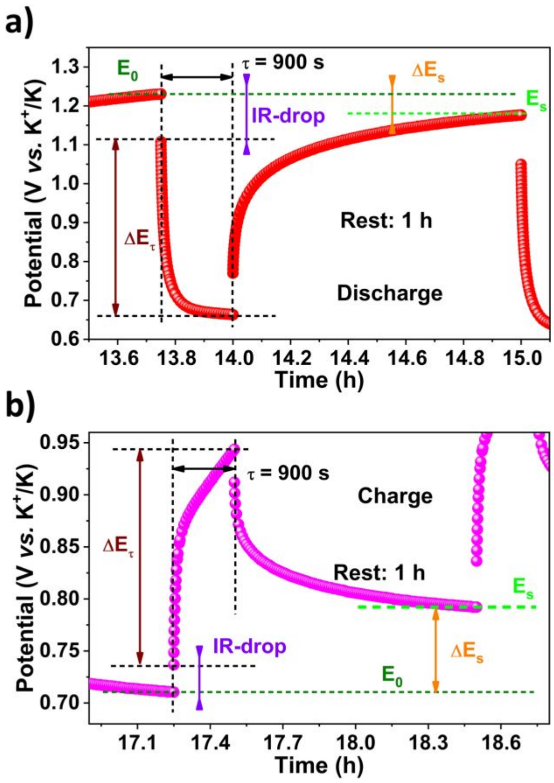

Figure S12. Parameter determination of GITT curve during (a) Discharge, and (b) Charge.

The K^+ diffusion coefficient during first potassiation and de-potassiation was obtained using Galvanostatic Intermittent Titration Technique (GITT), and this may be used to infer the K^+ diffusion kinetics in the Sb/CNT-2, compared with the pure Sb. Using Fick's second

law of diffusion, the diffusion coefficient of K^+ in the electrode is calculated based on Equation (S1) as follows:[5-7]

$$D_k = \frac{4}{\pi\tau}\left(\frac{m_B V_m}{M_b S}\right)^2\left(\frac{\Delta E_s}{\Delta E_\tau}\right)^2 \qquad \text{S1}$$

where τ is the time of applying a Galvanostatic pulse; m_B is the active mass in the anode; V_m and M_b are the molar volume and molar mass of the active material, respectively; S represents the geometry area of the electrode; and ΔE_s and ΔE_τ are obtained from GITT curve as explained in Figure S12.

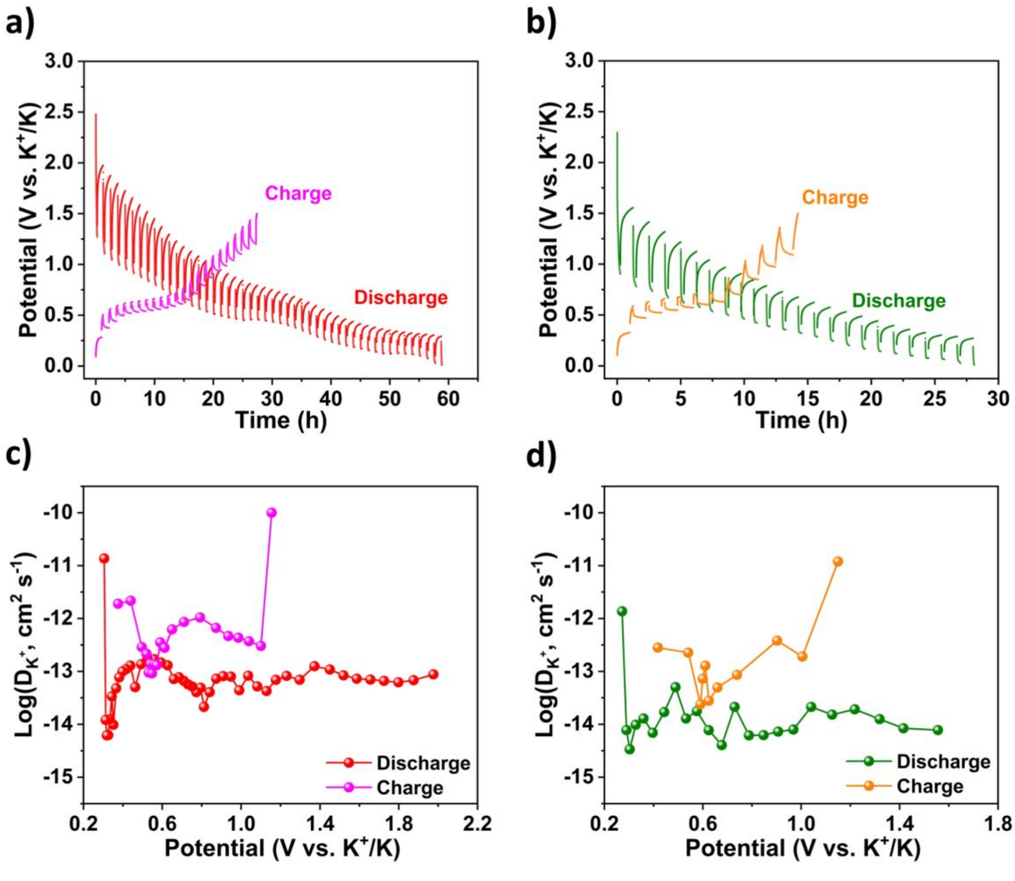

Figure S13. GITT curves for (a) the Sb/CNT-2 and (b) the pure Sb in the potential range of 0.01–1.5 V at a charge/discharge rate of C/5 for each pulse for 15 min and following

relaxation for 1 hour. And the calculated K^+ diffusion coefficient of (c) the Sb/CNT-2 and (d) the pure Sb tested at the first potassiation and de-potassiation.

The K^+ diffusion coefficient (D_{K+}) was calculated using Equation S1 from GITT graphs of the Sb/CNT-2 and the pure Sb (Figure. S14a, b). This data was plotted in Figure S14c (Sb/CNT-2) and Figure S14d (Sb). The D_{K+} in the Sb/CNT-2 showed values ranging from 9.54×10^{-15} to 1.38×10^{-11} $cm^2\ s^{-1}$ and 9.33×10^{-14} to 10.23×10^{-11} $cm^2\ s^{-1}$ for the discharge and charge processes, respectively. The results clearly show that the D_{K+} in Sb/CNT-2 was significantly higher than that in pure Sb, with D_{K+} in the values varying from 3.38×10^{-15} to 1.39×10^{-12} $cm^2\ s^{-1}$ during the discharge phase and from 2.39×10^{-14} to 1.9×10^{-11} $cm^2\ s^{-1}$ during the charge process. This implies that in terms of K^+ diffusion in the bulk active electrode material, Sb/CNT-2 is far more advantageous than pure Sb. As a result, the Sb/CNT-2 electrode may perform better electrochemically, compared to the pure Sb electrode.

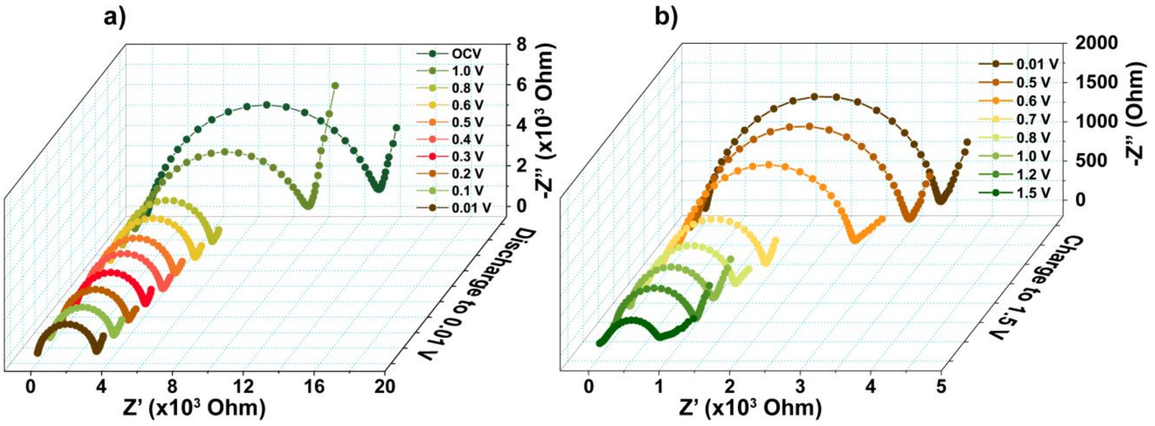

Figure S14. Ex situ EIS of the Sb/CNT-2 anode during the first discharge (a) and charge processes (b).

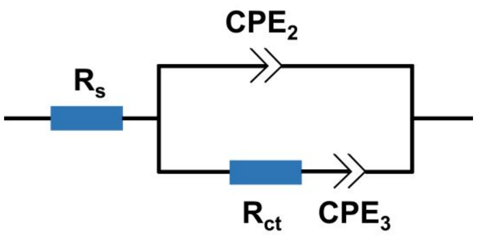

Figure S15. An equivalent circuit used for fitting of EIS data of the cell before cycling (without the SEI interface-layer element).

Table S3. The values of R_{ct} and R_{SEI} of Sb/CNT-2 obtained by circuit simulation based on ex-situ EIS.

Discharge voltage (V)	OCV	1.0	0.8	0.6	0.5	0.4	0.3	0.2	0.1	0.01
R_{ct} (Ω)	12595	5022	4646	4022	3743	3775	3665	3513	3319	3125
R_{SEI} (Ω)	-	4539	516	298.8	293.4	245.6	214.5	207.3	195.1	187.4

Charge voltage (V)	0.5	0.6	0.7	0.8	1.0	1.2	1.5
R_{ct} (Ω)	2772	2278	1368	1261	819	757.1	616.6
R_{SEI} (Ω)	184.4	155.2	148.4	142.4	137.8	135.8	130.4

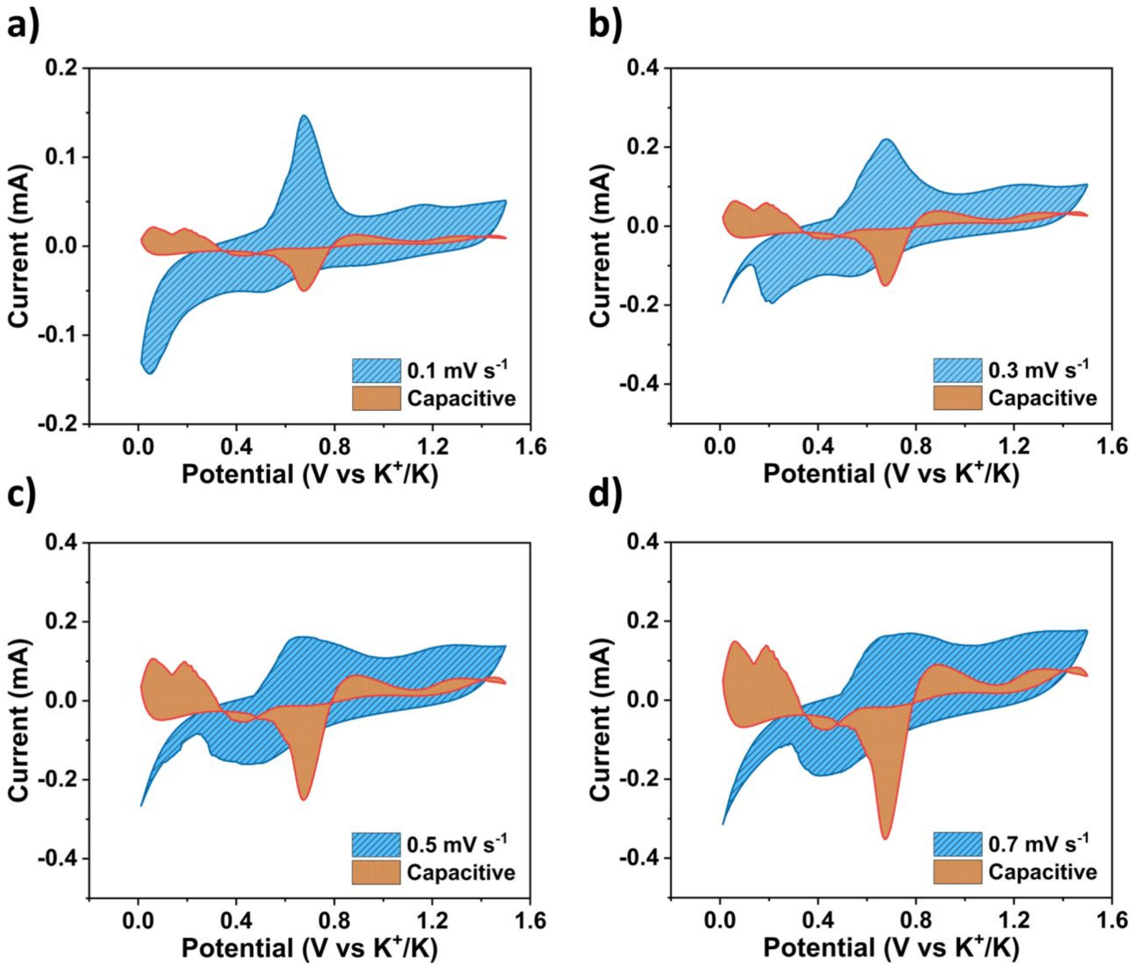

Figure S16. (a-d) Capacitance contribution of Sb/CNT-2 anode scanned at various rates.

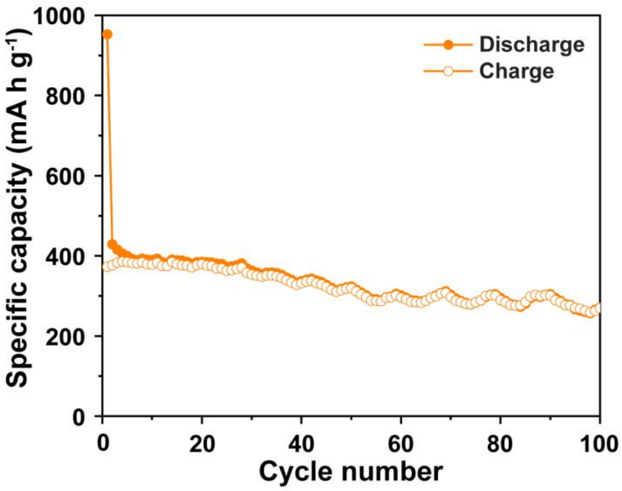

Figure S17. Cyclability of Sb/CNT-2 electrode with high mass-loading of 1.6 mg cm^{-2}, measured at a rate of C/5 (~132 mA g^{-1}).

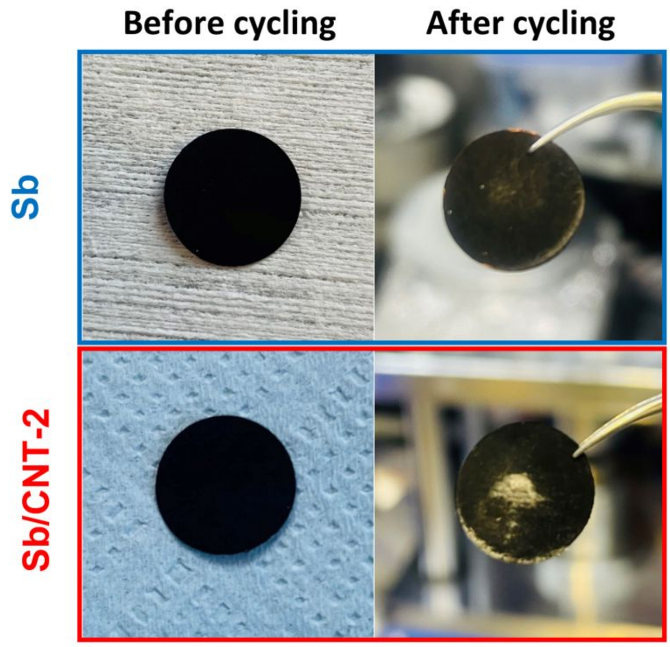

Figure S18. Optical images of Sb and Sb/CNT-2 electrodes before and after cycling.

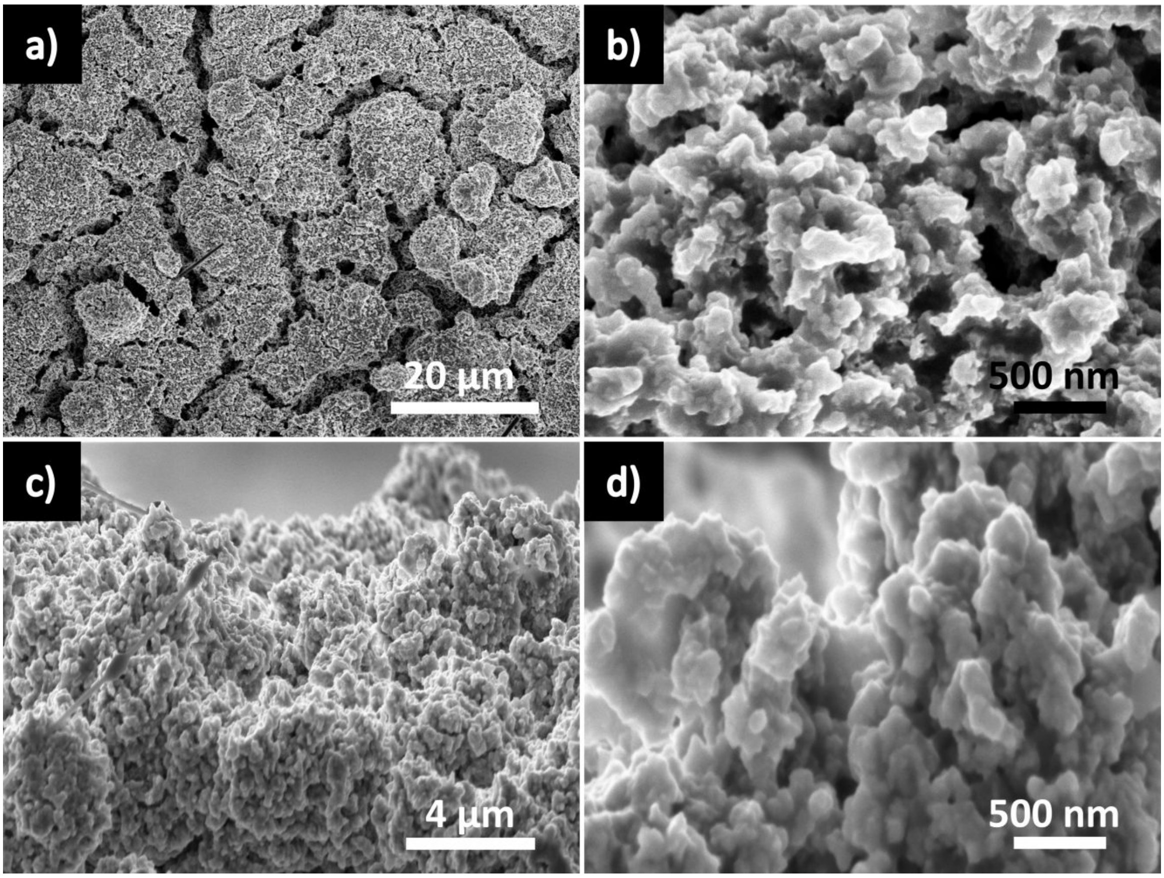

Figure S19. (a, b) Top-down SEM images and (c, d) cross-section SEM images of Sb electrode after the 100th cycle.

REFERENCES

(1) Zhang, W.; Chen, J.; Swiegers, G. F.; Ma, Z.-F.; Wallace, G. G. Microwave-Assisted Synthesis of Pt/CNT Nanocomposite Electrocatalysts for Pem Fuel Cells. *Nanoscale* **2010**, *2* (2), 282-286.

(2) Le, H. T. T.; Pham, X.-M.; Park, C.-J. Facile Citrate Gel Synthesis of an Antimony–Carbon Nanosponge with Enhanced Lithium Storage. *New J. Chem.* **2019**, *43* (27), 10716-10725.

(3) Pham, X.-M.; Ngo, D. T.; Le, H. T. T.; Didwal, P. N.; Verma, R.; Min, C.-W.; Park, C.-N.; Park, C.-J. A Self-Encapsulated Porous Sb–C Nanocomposite Anode with Excellent Na-Ion Storage Performance. *Nanoscale* **2018**, *10* (41), 19399-19408.

(4) Liang, S.; Cheng, Y.-J.; Wang, X.; Xu, Z.; Ma, L.; Xu, H.; Ji, Q.; Zuo, X.; Müller-Buschbaum, P.; Xia, Y. Impact of CO_2 Activation on the Structure, Composition, and Performance of Sb/C Nanohybrid Lithium/Sodium-Ion Battery Anodes. *Nanoscale Adv.* **2021**, *3* (7), 1942-1953.

(5) Imtiaz, S.; Kapuria, N.; Amiinu, I. S.; Sankaran, A.; Singh, S.; Geaney, H.; Kennedy, T.; Ryan, K. M. Directly Deposited Antimony on a Copper Silicide Nanowire Array as a High-Performance Potassium-Ion Battery Anode with a Long Cycle Life. *Adv. Funct. Mater.* **2023**, *33* (2), 2209566.

(6) Ngo, D. T.; Le, H. T. T.; Kim, C.; Lee, J.-Y.; Fisher, J. G.; Kim, I.-D.; Park, C.-J. Mass-Scalable Synthesis of 3D Porous Germanium–Carbon Composite Particles as an Ultra-High Rate Anode for Lithium Ion Batteries. *Energy Environ. Sci.* **2015**, *8* (12), 3577-3588.

(7) Luo, Y.; Liu, J.; Zhang, L. A Monocrystalline Coordination Polymer with Multiple Redox Centers as a High-Performance Cathode for Lithium-Ion Batteries. *Angew. Chem. Int. Ed.* **2022**, *61* (38), e202209458.
